# Impact of A134 and E218 Amino Acid Residues of Tropomyosin on Its Flexibility and Function

**DOI:** 10.3390/ijms21228720

**Published:** 2020-11-18

**Authors:** Marina A. Marchenko, Victoria V. Nefedova, Daria S. Yampolskaya, Galina V. Kopylova, Daniil V. Shchepkin, Sergey Y. Bershitsky, Natalia A. Koubassova, Andrey K. Tsaturyan, Dmitrii I. Levitsky, Alexander M. Matyushenko

**Affiliations:** 1 A.N. Bach Institute of Biochemistry, Research Center of Biotechnology, Russian Academy of Sciences, 119071 Moscow, Russia; marchenko_m@mail.bio.msu.ru (M.A.M.); viktoriya-neff@mail.ru (V.V.N.); daria_logvinova@mail.ru (D.S.Y.); levitsky@inbi.ras.ru (D.I.L.); 2 Department of Biochemistry, School of Biology, Moscow State University, 119234 Moscow, Russia; 3 Institute of Immunology and Physiology, Russian Academy of Sciences, 620049 Yekaterinburg, Russia; g_rodionova@mail.ru (G.V.K.); d.shchepkin@iip.uran.ru (D.V.S.); serg.bersh@gmail.com (S.Y.B.); 4 Institute of Mechanics, Moscow State University, 119192 Moscow, Russia; natalia@imec.msu.ru (N.A.K.); tsat@imec.msu.ru (A.K.T.)

**Keywords:** muscle contraction, tropomyosin, actin filaments, protein stability, differential scanning calorimetry, molecular dynamics

## Abstract

Tropomyosin (Tpm) is one of the major actin-binding proteins that play a crucial role in the regulation of muscle contraction. The flexibility of the Tpm molecule is believed to be vital for its functioning, although its role and significance are under discussion. We choose two sites of the Tpm molecule that presumably have high flexibility and stabilized them with the A134L or E218L substitutions. Applying differential scanning calorimetry (DSC), molecular dynamics (MD), co-sedimentation, trypsin digestion, and in vitro motility assay, we characterized the properties of Tpm molecules with these substitutions. The A134L mutation prevented proteolysis of Tpm molecule by trypsin, and both substitutions increased the thermal stability of Tpm and its bending stiffness estimated from MD simulation. None of these mutations affected the primary binding of Tpm to F-actin; still, both of them increased the thermal stability of the actin-Tpm complex and maximal sliding velocity of regulated thin filaments in vitro at a saturating Ca^2+^ concentration. However, the mutations differently affected the Ca^2+^ sensitivity of the sliding velocity and pulling force produced by myosin heads. The data suggest that both regions of instability are essential for correct regulation and fine-tuning of Ca^2+^-dependent interaction of myosin heads with F-actin.

## 1. Introduction

Tropomyosin is one of the major actin-binding proteins of the actin cytoskeleton [1]. Tpm was found in organisms of two kingdoms: animal and fungi, and its isoforms are selectively expressed in different tissues of many species [2]. Tpm is similar to scaffolding proteins and works like a ‘gatekeeper’ [3], determining partners for actin [2,4,5,6,7].

In cardiac and skeletal muscles, Tpm acts as a link between Ca^2+^-sensing proteins and those producing mechanical force [8,9]. Tpm molecules form a continuous strand, binding each other in a head-to-tail manner that can slide on the surface of the actin filament. These features, as well as interactions with other actin-binding partners—troponin complex and myosin—make it essential for the regulation of muscle contraction [8]. Tpm moves between three positions on actin surface: blocked—Tpm prevents the interaction between myosin heads and actin; closed—Tpm allows weak interaction of myosin with actin; open—myosin-binding sites on actin are available [9,10]. Troponin complex and myosin participate in Tpm transitions between the three states: troponin fixes Tpm in the blocking position on actin in Ca^2+^ free state; after Ca^2+^ binding and structural rearrangements, troponin allows blocked-closed Tpm transition [11]. Myosin, in its turn, induces Tpm movement from the closed position to the open state and opens neighboring actin sites for myosin binding [12]. MacKillop and Geeves offered this simple model of muscle regulation in 1993, and it is still relevant [13]. However, biophysical details of this process, as well as functioning in the pathogenic conditions, are not well understood. Particularly, an open question is the role of the Tpm molecule rigidity [14].

Although Tpm has a relatively simple structure, it performs many functions in the cell [15]. Tpm has a typical coiled-coil structure, which is well determined by its primary sequence [8]. The entire sequence can be divided into seven-part amino acid repeats—heptads [16]. Latin letters assign each amino acid in heptad from **a** to **g**. Usually, in **a** and **d** positions, it possess sizeable hydrophobic amino acid residues that stick together the hydrophobic core of two neighboring α-helices. In **e** and **g** positions, there are charged amino acid residues that form electrostatic bonds between α-helices and additionally stabilize the coiled-coil structure. In **b**,**c,** and **f** positions, can be polar or charged amino acids needed for the interaction of Tpm with partner proteins, especially actin [17]. If all amino acid residues in Tpm obeyed this rule, the Tpm molecule would be an ideal coiled-coil. However, many amino acid residues in the Tpm molecule brake this rule and lead to the appearance of curves, gaps, and sites of instability in the coiled-coil structure [18].

As it has been shown, the Tpm molecule has a semi-rigid structure and several regions of instability allow its optimal binding to actin filaments and effective mechanical transmission of Ca^2+^ activation signal along actin filaments [18,19,20]. These sites can be divided into two groups, with increased and reduced stability. Minakata and co-authors distinguished six alanine clusters and three breaks in the hydrophobic core as zones with potentially high flexibility [21]. There are two conserved charged amino acid residues D137 and E218 inside these zones that participate in the exposure of the hydrophobic core to the solvent and lead to the formation of the holes in the coiled-coil structure. This finding prompted us to pay attention to these two regions.

Instability in the middle part of Tpm was suspected long ago. Some of the first works in this area were devoted to the analysis of Tpm proteolysis products by trypsin [22,23]. It turned out that the primary site of proteolysis was located after Arg133 in the middle part of the Tpm molecule, suggesting that this part of the molecule is unstable [24]. The structure of the Tpm central part was published in 2005 [25]. In this work, the authors were able to find zones of loose packaging of the Tpm molecule in its central part leading to the formation of cavities [25]. The formation of such cavities is caused by the presence in this area of large residues of the Met, Ile, and Leu residues, as well as the charged Asp137 residue in the hydrophobic core and Ala residues that are unable to form hydrophobic density between two α-helices [25]. After the structure was published, the central part of Tpm was subjected to intensive functional studies. In some studies, two amino acids that destabilize the central part structure G126 and D137 were changed to R126 and L137 for functional studies [26,27]. These substitutions stabilzsed the central part of Tpm and altered its function: (i) increased ATPase activity of myosin subfragment 1 in the presence of reconstructed thin filaments and (ii) altered Ca^2+^-sensitivity of actin-myosin interaction [27,28,29].

Nevertheless, it is still unclear whether these functional changes were related to the direct interaction of Tpm with the myosin head or were caused by the alterations in the stiffness of the Tpm strand. To answer this question, we substitute conserved A134 residue with Leu in the Tpm structure (A134L). This substitution does not change the Tpm charge that can affect the interaction of Tpm with myosin or actin. Introduction of a Leu residue should close the central gap and stabilize the Tpm structure.

Another locus of instability is located near the E218 non-canonic residue in the C-terminal part of the Tpm molecule. However, this structural segment is much less studied. Two published structures suggest that this segment contains a hole between two α-helices [21,30]. It was shown that ∆E218 in Tpm3.12 increased the calcium sensitivity of the actin-myosin interaction and led to congenital fiber type disproportion [31]. Here, we introduced an E218L substitution and studied the structural and functional effects of this substitution.

It was assumed that these two regions are necessary for proper Tpm binding to F-actin and functioning [25,32,33]. So, in our work, we tried to identify the role of two islands of instability in the structure of Tpm molecule—the central part and C-terminal region near the E218 residue, to check whether these regions are necessary for correct Tpm binding to actin or effective mechanical transmission of the calcium signal along the Tpm strand.

## 2. Results

### 2.1. Stability of the A134L and E218L Tpm Molecules

We used a well-tested approach to study the functional significance of two regions in Tpm structure and replaced non-canonical amino acid residues (that presumably destabilize coiled-coil structure) to canonical ones introducing the A134L and E218L substitutions in the Tpm primary sequence. First of all, we estimated the stabilizing effect of these substitutions on the Tpm structure, applying two methods: limited proteolysis by trypsin and differential scanning calorimetry (DSC).

**Figure 1:**
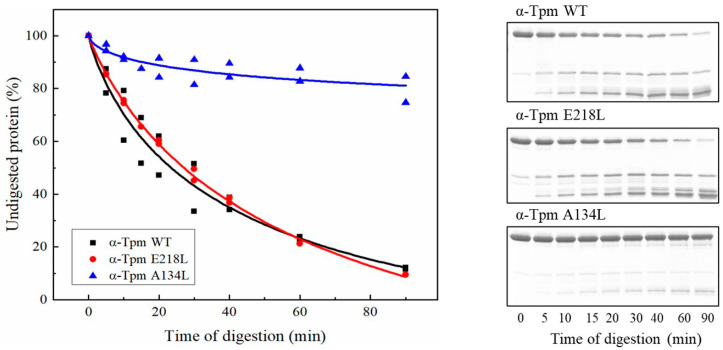
SDS-PAGE gels (**right**) and the time course of tryptic digestion (**left**) of A134L and E218L tropomyosin (Tpm) in comparison with WT Tpm. Trypsin:Tpm weight ratio was 1:150. Tpm concentration was 0.5 mg/mL; the reaction was carried out at 30 °C in 30 mM Hepes-Na buffer, 100 mM NaCl, 1mM MgCl_2_, pH 7.3.

**Figure 2:**
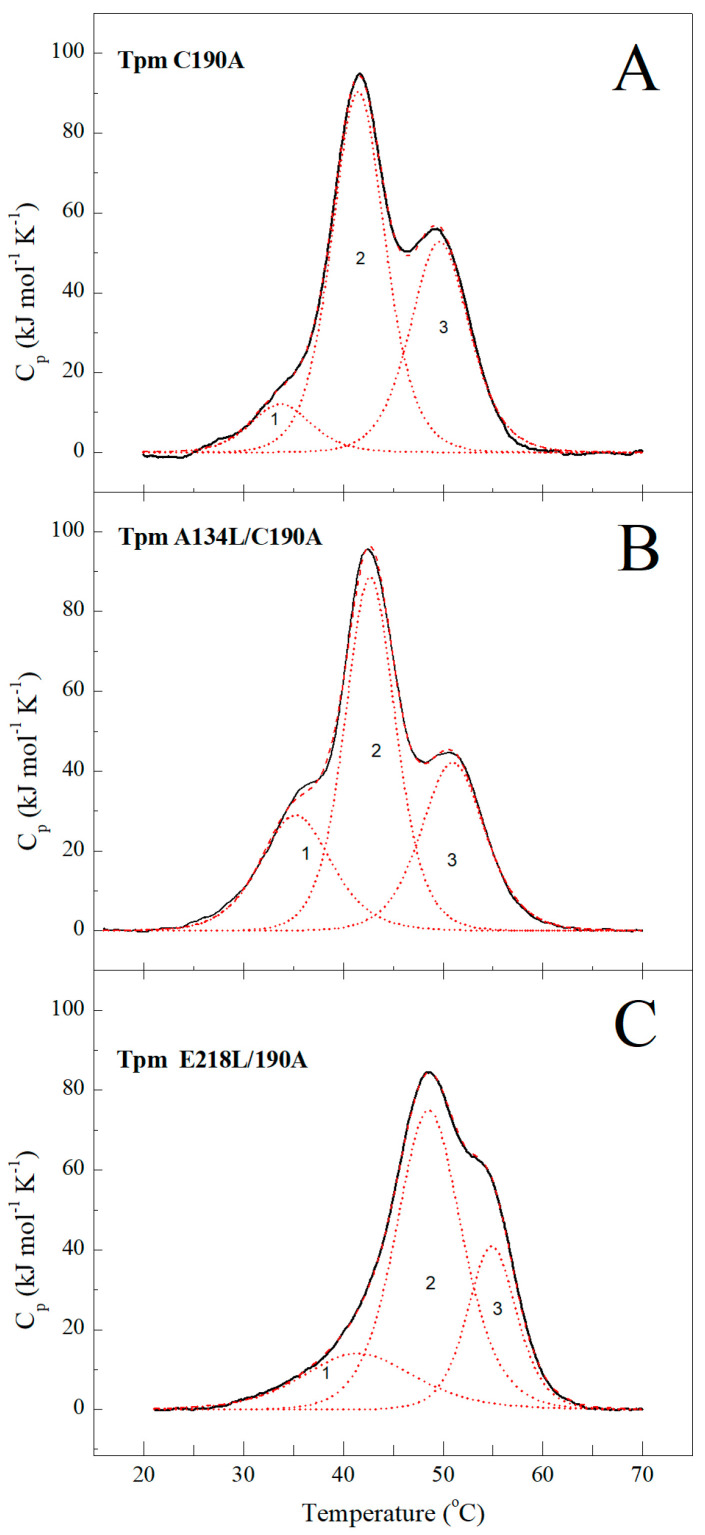
Heat sorption curves and deconvolution analysis of DSC profiles for A134L/C190A (**B**) and E218L/C190A (**C**) mutants in comparison with control C190A Tpm (**A**). Solid lines represent the experimental curves after subtraction of chemical and instrumental baselines, dotted curves represent the calorimetric domains obtained from experimental data by analysis with the non-two-state model. The experiments were done at a constant heating rate of 1 K/min and protein concentration 2 mg/mL.

The results of limited proteolysis showed that the A134L mutation almost entirely prevented the proteolysis of the Tpm by trypsin (Figure 1). At the same time, the A134L substitutions only slightly increased the thermal stability of Tpm (Figure 2, Table 1). The results indicate that A134L mutation stabilizes the central part of the Tpm molecule without significant effects on the whole Tpm structure. The E218L substitution did not affect the Tpm trypsinolysis (Figure 1) although significantly stabilized the Tpm structure as shown in DSC profiles in comparison with the A134L and control C190A Tpm molecules (Figure 2, Table 1). The 2nd and 3rd calorimetric domains of E218L Tpm were more thermostable than those of C190A Tpm (Figure 2, Table 1). The results of limited proteolysis of the E218L Tpm mutant is explained by the fact that the Tpm proteolysis occurs between the residues Arg133 and Ala134 of the molecule. 

### 2.2. Functional Studies

#### 2.2.1. Co-Sedimentation Assay and Thermal Stability of Tpm-F-actin Complexes

**Figure 3:**
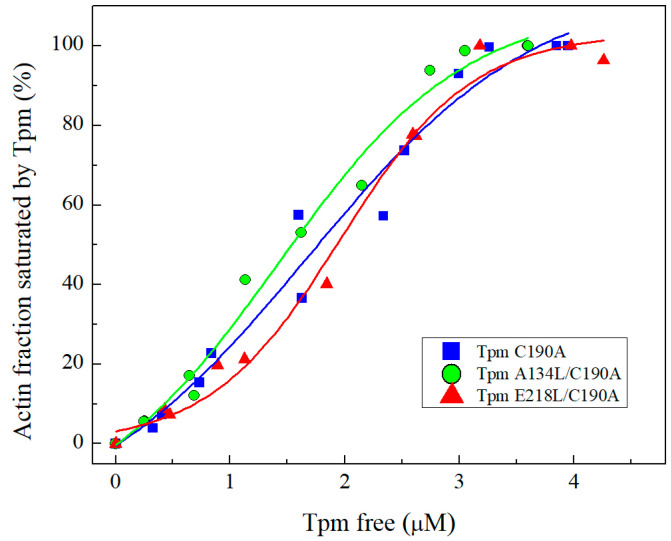
The affinity of Tpm species A134L/C190A, E218L/C190A, and control C190A Tpm for F-actin determined by co-sedimentation assay. The Tpm concentration of F-actin half-saturation (K_50%_) was 1.42 ± 0.18 μM for A134L/C190A, 1.96 ± 0.11 μM for E218L/C190A, and 1.68 ± 0.32 μM for C190A Tpm species. The K_50%_ values are the average ± SD for three experiments. All experimental points were plotted.

Both mutations did not significantly affect the Tpm affinity for actin determined with the co-sedimentation assay (Figure 3). The K50%, the parameter corresponding to the Tpm concentration at which F-actin is half-saturated, for the studied proteins was 1.42 ± 0.18 μM for A134L, 1.96 ± 0.11 μM for E218L, and 1.68 ± 0.32 μM for C190A Tpm. 

**Figure 4:**
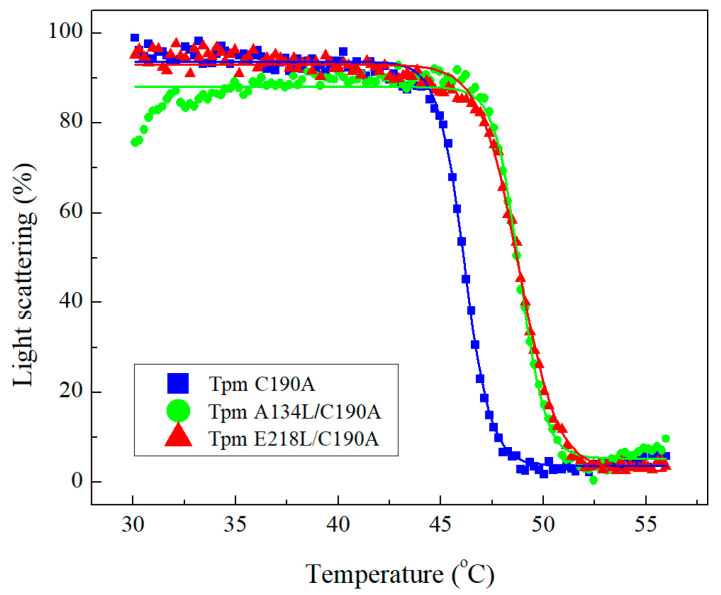
Normalized temperature dependencies of light scattering obtained for F-actin complexes with A134L/C190A, E218L/C190A, and C190A Tpm species. The standard experiment was done using 20 μM F-actin stabilized by an equal molar amount of phalloidin and 10 μM of Tpm in 30 mM Hepes-Na buffer containing 100 mM NaCl, pH 7.3. The decrease in light scattering reflects the dissociation of Tpm from F-actin.

However, the A134L and E218L mutation similarly increased the thermal stability of actin-Tpm complexes (Figure 4, Table 1). The temperature of dissociation, Tdiss (i.e., the temperature at which a 50% decrease in the light scattering occurs) provides valuable information about the stability of the Tpm-F-actin complex. The Tdiss for both Tpm mutants increased by ~2 °C compared to C190A control Tpm (Figure 4, Table 1) meaning that the F-actin complexes with both mutant Tpms are more thermostable than that with the C190A control Tpm. These data indicate that the A134L and E218L Tpm mutations did not affect the primary binding of Tpm to F-actin, although stabilized the Tpm interaction with F-actin compared to C190A Tpm.

#### 2.2.2. In Vitro Motility Assay

**Figure 5:**
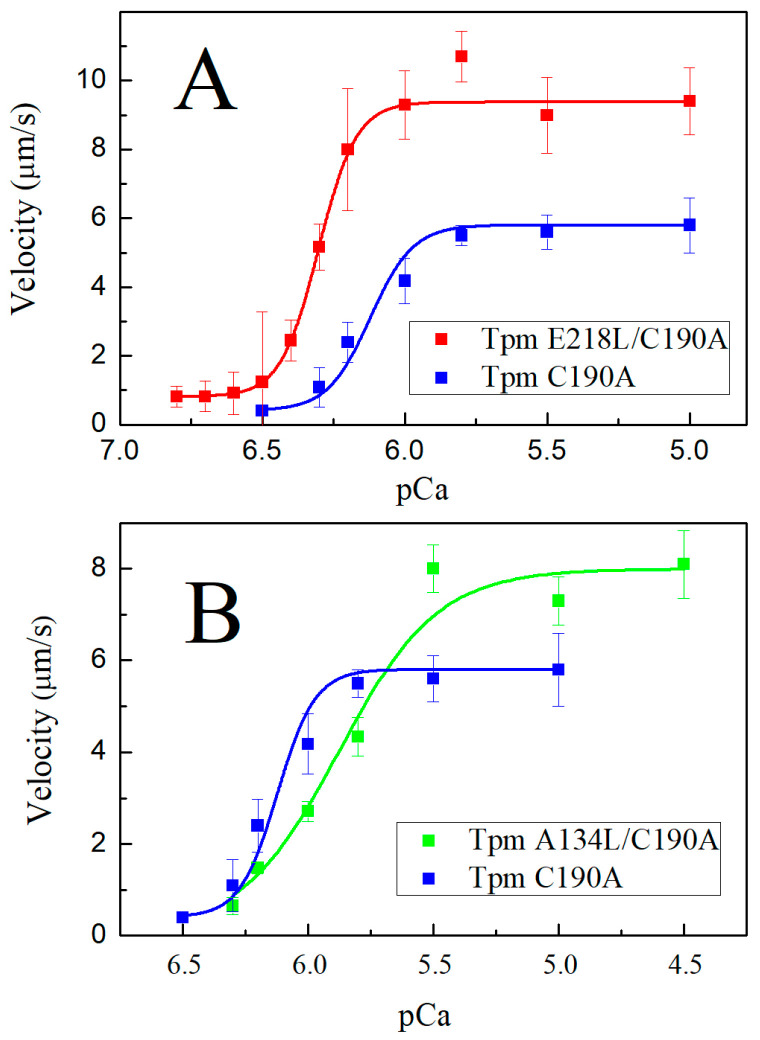
Ca^2+^-dependent sliding velocities of thin filaments containing E218L/C190A (**A**) and A134L/C190A (**B**) Tpm mutants in comparison with C190A Tpm obtained in the in vitro motility assay. Each data point represents the mean ± SD from three independent experiments. Data were fitted with the Hill equation as described in Material and Methods. The fitting parameters are shown in Table 2.

We also investigated the effect of A134L and E218L substitutions on the velocity of reconstructed thin filaments in the in vitro motility assay (Figure 5). Both mutations increased the maximum sliding velocity at saturating Ca^2+^concentrations. The velocity was 1.6 times higher for the E218L Tpm mutant and 1.4 times higher for the A134L one than that for the C190A control Tpm (Table 2). However, the effects on Ca^2+^ sensitivity of thin filament sliding velocity were opposite for these two mutations (Figure 5, Table 2). The E218L substitution increased the Ca^2+^ sensitivity of the sliding velocity while the A134L one decreased it (Table 2).

**Figure 6:**
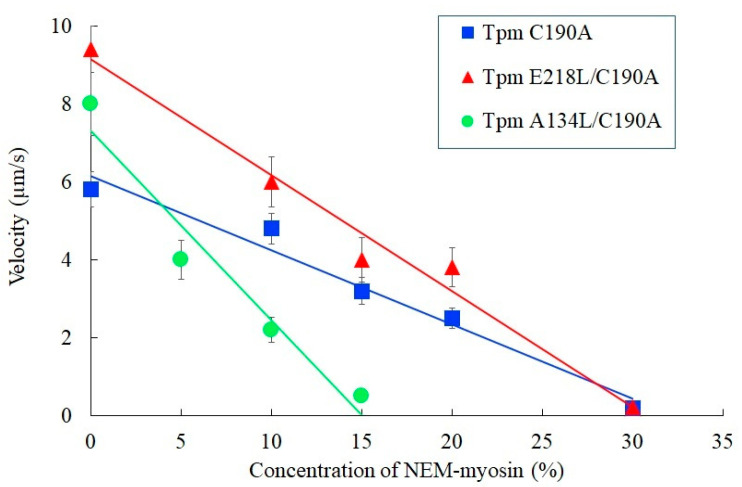
The dependence of the sliding velocity of thin filaments containing A134L/C190A, E218L/C190A, and C190A Tpm on the NEM-modified myosin concentration in the in vitro motility assay at *p*Ca 4. The fraction of NEM-myosin required to stop the filament movement is a measure of relative force, which myosin can develop. Each data point represents the mean ± SD of three experiments. To stop the movement of thin filaments with C190A Tpm, A134L/C190A Tpm, and E218L/C190A Tpm, 32 ± 4%, 15 ± 2%, and 31 ± 3% of NEM-myosin were needed, respectively.

The other exciting results were obtained in experiments with different concentrations of NEM-modified myosin that slows or stops the sliding velocity in the motility assay (Figure 6). This test gives an estimate of the force-generating capacity of myosin molecules during their interaction with thin filaments, as the heads of NEM-treated myosin bind actin in an ATP-independent manner and serve as load. For the A134L mutant, the relative force developed by myosin heads in the motility assay decreased by a factor of 2 compared to control C190A Tpm, while the E218L one did not affect it. The data obtained using in vitro motility assay showed that both mutations alter parameters of actin–myosin interaction, indicating that naturally occurring instability in these Tpm regions is essential for correct calcium regulation of actin–myosin interaction. However, these mutations lead to the different effects observed in such type experiments.

### 2.3. MD Simulations

**Figure 7:**
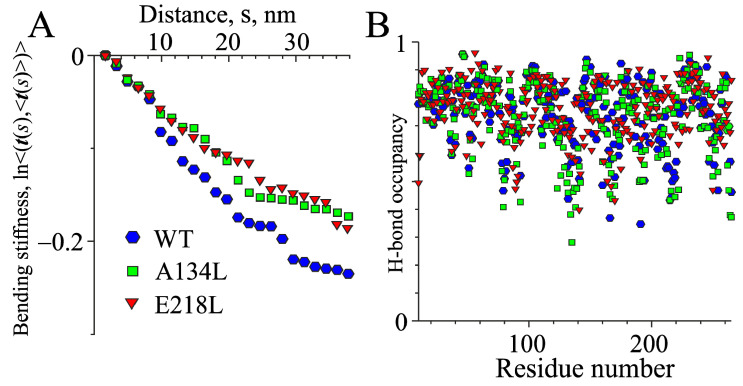
The result of the molecular dynamics (MD) simulation of the WT Tpm and the Ala134Leu and Glu218Leu Tpm mutants. Left: characteristic of the bending stiffness (see Materials and Methods section, less steep dependency corresponds to higher stiffness) (**A**); right: the occupancies of the backbone *h*-bonds (**B**).

It should be noted that the Protein Data Bank (PDB) does not have a high-resolution structure of a complete Tpm molecule. The 1C1G structure has a resolution of only 0.7 nm, and the quality of this structure does not allow for its use for MD calculations. For this reason, a refined structure using higher resolution Tpm fragment structures (PDB entries 2D3E, 2EFR, 3AZD, 1KQL, 5KHT, and 2B9C) was prepared. The Tpm coiled-coil was skeletonized to evaluate the bending stiffness and time course of skeleton bending and was analyzed as described in Methods. The effects of amino acid substitutions A134L and E218L on the MD simulations are shown in Figure 7. The A134L substitution fills a ‘gap’ in the central part of the molecule with a larger (compared to Ala) hydrophobic Leu side chain. At the same time, the E218L one introduces a ‘canonic’ hydrophobic residue in the position **a** of the heptadic repeat of Tpm coiled-coil.

Both substitutions led to an increase in the bending stiffness, and, correspondingly, the persistent length of Tpm (Figure 7A). The values of the secant persistent length *L_p_* obtained from MD calculations (Figure 7A) were 161 nm for WT Tpm, 218 nm for A134L Tpm, and 203 nm for the E218L one. In terms of the Tpm bending stiffness, this corresponds to 660, 894, and 832 pN × nm^2^, respectively.

Both mutations increased the rigidity of the central and C-terminal regions of the molecule, not affecting the occupancies of hydrogen bonds systematically along the entire Tpm length except a significant stabilization of the backbone *h*-bond network in the vicinity of the residue 218 (Figure 7B). The results of the MD calculations were in good agreement with experimental data on Tpm stabilizations caused by both Tpm mutations demonstrating a cooperative mechanism of the transmission of the stabilizing effect of single amino acid substitutions to the distant parts of the molecule.

## 3. Discussion

Although Tpm has a simple coiled-coil structure, it performs multiple functions in the thin filament. In muscle, it is a part of Ca^2+^ regulatory mechanism, a stabilizer of the thin filament and, according to the findings, is involved in binding to F-actin, myosin, and troponin [34,35,36]. The mechanism of Tpm binding to actin and the detailed mechanism of Tpm shifting upon Ca^2+^ activation and/or myosin binding to F-actin is still poorly understood. The essential role in these processes is attributed to Tpm flexibility. Here, we investigated two conserved Tpm regions possessing an internal instability, which, according to the structural data, tend to form gaps in the Tpm structure. The key features of these regions are the presence of charged amino acids in the coiled-coil core and the availability of the hydrophobic core for the solvent. It was suggested in previous works, that these regions are necessary for the correct Tpm binding to actin [25,32,33]. To test the proposed hypothesis, we applied the co-sedimentation assay. It turned out that stabilization of Tpm by the A134L or E218L substitutions did not affect the primary binding of Tpm to actin (Figure 3). However, we cannot completely exclude the impact of these regions on Tpm binding to actin. The instability of these regions may be necessary for the transitions between the three states of Tpm on actin. Both stabilizing mutations, A134L and E218L, increased the thermal stability of the Tpm-actin complex (Figure 4). Changes in the calcium sensitivity of the actin–myosin interaction in the in vitro motility assay experiments (Figure 5, Table 2) also indicate that Tpm with a more stable central or C-terminal part may shift the equilibrium towards one of the three states.

The A134L and E218L mutations stabilized the Tpm molecule: the A134L one prevented trypsinolysis, and both A134L and E218L substitutions suppressed the thermal denaturation (Figure 1 and Figure 2, Table 1). Our MD simulation suggested that both mutations increased the bending stiffness of the whole Tpm molecule (Figure 7). In turn, a change in the stiffness should lead to an alteration in the Tpm functional properties and affect the activation of contraction [36]. These changes are visible in the experiments using the reconstructed thin filament in the in vitro motility assay. Both substitutions increased the maximum velocity of thin filaments but differently affected Ca^2+^-sensitivity of the actin–myosin interaction. The increase in the velocity can be explained by the Tpm stiffening that increases the length of the cluster of myosin-binding sites on F-actin, which become open upon strong binding of a myosin head. As a result, the open state becomes over open.

Although the A134L Tpm mutation increased the sliding velocity of thin filaments at saturating Ca^2+^ concentration, it decreased the force produced by myosin heads two-fold compared to that of C190A Tpm and the E218L Tpm (Figure 6). Besides, the A143L substitution decreased the Ca^2+^ sensitivity of the sliding velocity, while the E218L one increased it. The difference between the effects of the A134L and E218L substitutions on the Ca^2+^ sensitivity and the pulling force can be caused by several factors. Previously, we investigated the effect of two stabilizing mutations in the vicinity of the 134th Tpm residue and have suggested that they may alter the interaction of a myosin head with Tpm [28]. The G126R and D137L substitutions that change the electrostatic interactions between Tpm and myosin increased both the Ca^2+^ sensitivity and maximal sliding velocity of reconstructed thin filaments [28]. It was suggested that the G126R and D137L substitutions might promote the transition of actin-bound Tpm into the open state upon the myosin binding via changes in electrostatic interaction of Tpm with myosin head [28]. In contrast to G126R and D137L, the A134L mutation did not change the Tpm charge, although increased the stability of the central part of Tpm and the rigidity of its structure. Moreover, as seen from the structures of the F-actin–Tpm–troponin complex in the blocked and closed states [36] as well as the F-actin-Tpm-myosin complex [35], the side chains of the 134th Tpm residue do not participate in direct interaction with actin, myosin head, or troponin.

Possibly, the Tpm stiffening itself hampers the blocked-to-close transition of the Tpm strand, thus decreasing the calcium sensitivity of the actin–myosin interaction at sub-saturating Ca^2+^ concentrations (Figure 5B). A decrease in the Ca^2+^ sensitivity with the A134L mutation was also accompanied by a reduction in cooperativity of the *p*Ca-velocity relation (Figure 5B, Table 2). The decline in force produced by myosin heads, which pull thin filaments containing the A134L mutant Tpm compared to the filaments with control C190A Tpm (Figure 6) can also be caused by the reduction in the cooperativity that in turn results in a decrease in the linear density (number per a length unit) of myosin heads pulling the thin filament.

**Figure 8:**
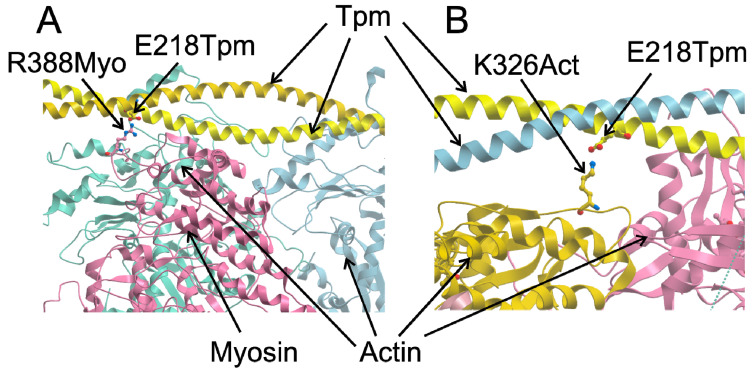
Structural models illustrating possible involvement of the Glu218 Tpm residue in the interaction with myosin (**A**) and actin (**B**). The models were obtained by minor side-chain modifications using a rotamer library with UCSF Chimera [37] from the structure of the F-actin–Tp–myosin complex in the open state (**A**, [35], PDB entry 4A7H) and the F-actin-Tpm complex in the closed state (**B**, [36], 6KN8).

With the E218L mutation, we observed an increase in the maximal sliding velocity of thin filaments and its Ca^2+^ sensitivity in the in vitro motility assay, the alterations similar to those with the G126R and D137L substitutions, which also changed charge of the Tpm molecule [28]. We hypothesized that the 218th Tpm residue might also be involved in the interaction with the myosin head. We tested this hypothesis by building a model using the structure of the actin–Tpm–myosin complex [35]. The model shows that the E218 Tpm residue can interact with the R388 residue of the myosin head in the open state (Figure 8A). Similar to the D137L substitution, a change in the electrostatic interaction between the head and Tpm caused by the E218L substitution can facilitate the displacement of Tpm to the open state upon myosin binding to actin. Another possible explanation of the effect of the E218L substitution on actin–myosin interaction in vitro is supported by the structure of the F-actin–Tpm–troponin complex in the blocked and closed states [36]. In these structures, the charged side chain of E218 Tpm residue could form an *h*-bond with the K326 residue of actin (Figure 8B). The substitution of the charged E218 residue with hydrophobic Leu should prevent the formation of the *h*-bond. Loss of the h-bonds along the whole Tpm strand may facilitate the transition to the open state of the regulated thin filament.

Our data provide a new example of the stabilization of the entire Tpm molecule caused by the introduction of single amino acid substitutions in different parts of the molecule. Both A134L and E218L mutations stabilized the whole Tpm structure, although at various degrees, as was observed with DSC (Figure 2) and simulated with MD (Figure 7). These results indicate high cooperativity of alterations in the Tpm structure; in other words, a mutation in one region can lead to changes in distant areas of the molecule. It is worth noting that similar results were also obtained earlier [38,39,40]. The possibility that a single substitution in the primary sequence of Tpm can lead to global changes in the structure opens a new perspective in investigations of the development of hereditary myopathies caused by point mutations in Tpm. Thus, the pathogenic effects of mutations can be caused not only by the local changes in structure but also be associated with the changes in the properties of sites located at a significant distance from the point mutation.

## 4. Materials and Methods

### 4.1. Protein Preparations

All Tpm constructs were obtained in the pMW 172 vector. C190A Tpm constructs were obtained in previous works. A134L and E218L Tpm mutants were cloned by site-directed PCR mutagenesis using Q5 site-directed mutagenesis kit (NEB, Ipswich, MA, USA, E0554). The pair of primers used for PCR procedures were GAGTCGA**CTC**CAAAAAGATGAAG—as a forward primer and TCAATGACTTTCATGCCTCTCTC—as an adjacent primer for A134L; CGCAGAAG**CTA**GACAGATATGAGG—as a forward primer and AGTACTTCTCAGCCTGAGCCTCC—as an adjacent primer for E218L. All constructs were sequenced to verify substitutions. All coding sequences were cloned between the NdeI and EcoRI restriction sites. The coding sequences of Tpm were preceded by the 5′-GCTAGC-3′ corresponding to the Ala-Ser dipeptide, imitating the naturally occurring N-terminal acetylation, which is necessary for the interaction of Tpm with actin [41]. As it has been shown, Tpm exists in the cell in a reduced state [42,43]. To avoid Tpm reducing, we used C190A Tpm substitution as a WT control. As published earlier, this mutation did not affect Tpm properties and well mimicked the fully reduced state of the molecule [27,44].

All Tpm species were expressed in E. coli C41 (DE3) in overnight cultures. Expression was induced by adding 1 mM IPTG at an optical density of 0.6—0.7. Tpm extraction and purification were performed as described previously [28]. The purified proteins were stored at −80 °C. All biochemical and biophysical experiments were performed on Tpm in the presence of 2 mM DTT.

Actin extraction and purification from *m. psoas* of the rabbit was carried out according to standard protocols [45]. F-actin was further stabilized by adding phalloidin in a molar ratio of 1:1. For the in vitro motility assay, F-actin was labelled with a 2-fold molar excess of TRITC-phalloidin (Sigma Chemical Co., St. Louis, MO, USA). The concentration of actin was measured spectrophotometrically at 290 nm using a molar extinction coefficient of 0.63 (mg/mL)^−1^ × cm^−1^.

Myosin and troponin were extracted from *m. psoas* of the rabbit by the standard methods [46,47]. 

All procedures involving animal care and handling were performed according to institutional guidelines set forth by Animal Care and Use Committee at the Institute of Immunology and Physiology Ural Branch of RAS and Directive 2010/63/EU of the European Parliament.

### 4.2. Trypsin Digestion 

L-1-tosylamido-2-phenylethyl chloromethyl ketone-treated trypsin (Worthington Biochemical Corp., Lakewood, NJ, USA) was added to 0.5 mg/mL Tpm samples in 30 mM Hepes-Na buffer, 100 mM NaCl, 1 mM MgCl_2_, pH = 7.3 at trypsin:Tpm weight ratio of 1: 150 at 30 °C. The reaction was stopped by adding SDS-PAGE sample buffer with 5 mM phenylmethanesulfonyl fluoride at different time intervals. Samples were subjected to SDS-PAGE electrophoresis; gels were scanned, and obtained images were analyzed by ImageJ 1.45 and Origin 9.0 software (OriginLab Corp., Northampton, MA, USA). 

### 4.3. Differential Scanning Calorimetry (DSC)

The heat sorption curves of Tpm were obtained on a MicroCal VP-Capillary differential scanning calorimeter (Malvern Instruments, Northampton, MA 01060, USA). All experiments were performed with 2 mg/mL protein concentration in 30 mM Hepes-NaOH buffer, 100 mM NaCl, pH 7.3 at a heating rate of 1K/min. The calorimetric traces were corrected for the instrumental background by subtracting a scan with the buffer in both cells. The temperature dependence of the excess heat capacity was further analyzed and plotted using Origin 7.0 software (MicroCal Inc., Northampton, MA, USA). The deconvolution procedure was carried out in the Origin 7.0 software using the algorithms described earlier [48,49].

### 4.4. Co-Sedimentation Assay

Tpm affinity to actin was determined using the co-sedimentation assay as described earlier [50,51]. Briefly, the probes contained 9.4 μM actin in 30 mM Hepes-Na, 200 mM NaCl, pH 7.3, and Tpm in the concentration ranging from 0.75 to 5 μM were incubated for 40 min at room temperature and centrifuged at 133,000× *g* for 40 min (Beckman Airfuge; Beckman Coulter, Fullerton, CA, USA). Samples were analyzed by SDS-electrophoresis. Gels were scanned and processed using ImageJ 1.45 and Origin 9.0 software packages to determine the amount of the proteins in the pellet and supernatant.

### 4.5. Temperature Dependences of Light Scattering

Thermally-induced dissociation of Tpm complexes with F-actin was determined from the change in light scattering measured at 352 nm at an angle of 90° on a Cary Eclipse spectrofluorimeter (Varian Australia Pty Ltd., Mulgrave, Victoria, Australia) equipped with a temperature controller as described earlier in [28,52]. A standard probe contained 20 μM F-actin stabilized with phalloidin and 10 μM Tpm in 30 mM Hepes-Na and 100 mM NaCl at pH 7.3. All measurements were performed at the heating rate of 1 °C/min. The temperature-dependent decrease in the light scattering intensity of the Tpm-F-actin complexes reflects dissociation of Tpm from F-actin. The scattering of F-actin solutions containing the same concentration of actin (20 μM) as that in the Tpm–F-actin samples was measured before the experiments and deducted from experimental curves. Impact of free Tpm molecules to the light scattering was negligible. The dissociation curves were analyzed by Origin software (MicroCal, Studio City, CA, USA) using Boltzmann sigmoidal decay function.

### 4.6. In Vitro Motility Assay 

Measurements of the sliding velocity of the regulated thin filaments at different Ca^2+^ concentrations were performed in the in vitro motility assay as described earlier [28,29]. In brief, myosin (300 µg/mL) in AB buffer (25 mM KCl, 25 mM imidazole, 4 mM MgCl_2_, 1 mM EGTA, and 20 mM DTT, pH 7.5) containing 0.5 M KCl was loaded into the experimental flow cell. After two minutes, 0.5 mg/mL BSA was added for 1 min. Further, 50 µg/mL of non-labelled F-actin in AB buffer with 2 mM ATP was added for 5 min to block nonfunctional myosin heads. Then, 10 nM TRITC-phalloidin labelled F-actin was added for five minutes. Unbound F-actin was washed out with AB buffer. Finally, the cell was washed with AB buffer containing 0.5 mg/mL BSA, oxygen scavenger system, 20 mM DTT, 2 mM ATP, 0.5% methylcellulose, 100 nM Tpm/Tn, and appropriate Ca^2+^/EGTA in proportions calculated with the Maxchelator program (https://somapp.ucdmc.ucdavis.edu/pharmacology/bers/maxchelator/downloads.htm). The experiments were done at 30 °C; the sliding velocity of the 30–100 filaments was measured using the GMimPro software (freeware applications written by G. Mashanov, The Crick Institute, UK) [53]. Experiments were repeated three times with each of the Tpm mutants, and the means of individual experiments were fitted to the Hill equation:

v = v_max_(1 + 10^n(*p*Ca-*p*Ca50)^)^−1^ (1)

where *v* and *v*_max_ are velocity and the maximal velocity at saturating calcium concentration, respectively; *p*Ca_50_ (i.e., calcium sensitivity) is *p*Ca at which half-maximal velocity was achieved; and *n* is the Hill coefficient. 

Effect of the Tpm mutants on the force of myosin interaction with regulated thin filaments was assessed using NEM-modified myosin as an external load [54,55]. The experimental protocol was similar to that described above except the blocking of nonfunctional myosin heads with non-labelled F-actin was omitted. A mixture of native and NEM-modified myosin at the total concentration of 100 µg/mL was used in the in vitro motility assay at saturating Ca^2+^ concentration instead of native myosin. We analyzed the dependence of the sliding velocity of thin filaments on the percent ratio of NEM-myosin to WT myosin added to the flow cell. The relative force was expressed as a percentage of NEM-myosin, which is required to stop the filament movement. It was estimated by linear extrapolation to zero velocity.

### 4.7. MD Simulation

The MD simulation was performed using GROMACS v. 2018.3 and v. 2019.3 [56]. The structure of full Tpm1.1 molecule (PDB code 1C1G, [37]) was used as a starting model. As the 1C1G structure was obtained for pig Tpm1.1, residue substitutions were made to build a human WT Tpm1.1 model. The substitutions, as well as the models of the Ala134Leu and Glu218Leu Tpm mutants, were built with the UCSF CHIMERA (University of California, San Francisco, California, USA) [57]. The model structures were immersed in a 10 × 10 × 45 nm rectangular box filled with TIP3P [58] water molecules with the addition of Na^+^ and Cl^−^ ions to provide zero net charge and the ionic strength of 0.15 M. The energy minimization, the equilibrations in the NVT and NPT ensembles, and 200-ns long MD runs was performed with the AMBER99SB-ILDN force field [59] as described earlier [60].

### 4.8. Analysis of MD Trajectories

The bending stiffness of the Tpm coiled-coil was estimated as described previously [51]. Briefly, the snapshots were recorded every 100 ps of each MD trajectory. The axis of the Tpm coiled-coil in each snapshot was approximated by a polygonal line using centroids of the C_α_ atoms of 11 consequent amino acids of each polypeptide chain (the first and 11th residues were taken with a weight factor of 0.5). The first segments of all lines were superimposed and the time average unit vectors ***t***_0_(*s*) = <***t***(*s*)> of each segment were calculated (*s* is the distance along the Tpm coiled-coil axis). Then ln<(***t***(*s*), ***t***_0_(*s*))> was plotted against *s*, ((***x***, ***y***) denotes the scalar product of vectors ***x*** and ***y***) assuming that <(***t***(*s*), ***t***_0_(*s*))> = exp(−*s*/*L*), *L = K**/**k_B_T*, where *L* is the persistence length proportional to the bending stiffness *K, k_B_* is the Boltzmann constant, and *T* is the absolute temperature. This approach is a generalization of the worm-like theory [60] for a semi-rigid rod with a priori unknown intrinsically bent shape [51]. The occupancy of the backbone hydrogen bonds (*h*-bonds) was calculated with a Python script using the *hbond* function of GROMACS. The average occupancy values for identical residues of both Tpm chains were plotted against the residue number to characterize the α-helix stability along the molecule.

## 5. Conclusions

In the Tpm molecule, there are several regions with increased flexibility. The role of these regions is not well understood. Here, we have demonstrated that the modification of two islets of instability in the central and C-terminal parts of the Tpm molecule does not affect the primary binding of Tpm to actin filaments, though both mutations increase the rigidity of the molecule. This stabilization can lead to alterations in Ca^2+^-induced activation of the myosin–actin interaction and impair the proper regulation of muscle contraction.

## Figures and Tables

**Table 1:** Calorimetric parameters obtained from the differential scanning calorimetry (DSC) data for individual thermal transitions (calorimetric domains) of Tpm mutants and parameters of thermostability of the Tpm–F-actin complexes (T_diss_).

Tpm	T_m_^a^ (°C)	ΔH_cal_^b^ (Kj*mol^−1^)	Total ΔH_cal_^b^ (Kj*mol^−1^)	ΔH_cal_ (% of total)	T_diss_^c^ (°C)
C190A			1155		46.14 ± 0.02
domain 1	33.7	100		8.7	
domain 2	41.5	640		55.4	
domain 3	49.7	415		35.9	
A134L/C190A			1235		48.84 ± 0.04
domain 1	35.3	275		22.3	
domain 2	42.7	600		48.6	
domain 3	51.0	360		29.1	
E218L/C190A			1141		48.80 ± 0.03
domain 1	41.6	210		18.4	
domain 2	48.6	661		57.9	
domain 3	54.9	270		23.7	

^a^—The error of the transition temperature (T_m_) values did not exceed ± 0.2 °C; ^b^—The relative error of the calorimetric enthalpy values, ΔH_cal_, did not exceed ± 10%; ^c^—T_diss_ is the temperature of the half-maximal dissociation of Tpm-F-actin complexes, that is, the temperature at which a 50% decrease in the light scattering occurs; the T_diss_ values presented as mean ± SD for three experiments.

**Table 2:** Parameters of the Hill equation for the dependence of the sliding velocity of thin filaments containing Tpm mutants in the in vitro motility assay.

Tpm	V_max_, µm/s ± SD	*p*Ca_50_ ± SD	*n* ± SD
C190A	5.8 ± 0.2	6.13 ± 0.01	3.1 ± 0.2
A134L/C190A	8.0 ± 0.8	5.89 ± 0.07	1.3 ± 0.2
E218L/C190A	9.4 ± 0.1	6.30 + 0.01	3.5 + 0.3

Abbreviations: V_max_, the maximal sliding velocity of thin filaments measured at a saturating Ca^2+^ concentration (*p*Ca 5); *p*Ca_50_, *p*Ca at which the sliding velocity is half-maximal; *n*, Hill cooperativity coefficient.

## Abbreviations

Tpm	tropomyosin
F-actin	filamentous actin
DSC	differential scanning calorimetry
MD	molecular dynamics
*h*-bond	hydrogen bond
